# Dose reduction potential of using gold fiducial markers for kilovoltage image‐guided radiotherapy

**DOI:** 10.1002/acm2.13023

**Published:** 2020-09-22

**Authors:** Yoshiki Takei, Hajime Monzen, Mikoto Tamura, Hiroshi Doi, Yasumasa Nishimura

**Affiliations:** ^1^ Department of Medical Physics Graduate School of Medical Sciences Kindai University Osakasayama Japan; ^2^ Department of Radiology Kindai University Nara Hospital Ikoma Japan; ^3^ Department of Radiation Oncology Faculty of Medicine Kindai University Osakasayama Japan

**Keywords:** dose reduction, fiducial marker, kV IGRT

## Abstract

This study aimed to evaluate the possibility of reducing the imaging dose for image‐guided radiotherapy by using planar kilovoltage orthogonal imaging and fiducial markers (kV‐FM). We tested kilovoltage planar images under clinical imaging conditions for the pelvis (75 kVp, 200 mA, 50 ms) at a decreasing tube current (from 200 to 10 mA). Imaging doses were measured with a semiconductor detector. The visibility of the kV‐FM, aspects of image quality (spatial resolution, low contrast resolution), and the resultant image registration reproducibility were evaluated using various shapes (folded, linear, tadpole‐like) of fiducial markers containing 0.5% iron [Gold Anchor™ (GA); Naslund Medical AB, Huddinge, Sweden]. The GA phantom was created by placing these variously shaped GAs in an agar phantom. The imaging doses with 200 and 10 mA were approximately 0.74 and 0.04 mGy and they were correlated to the tube current (R^2^ = 0.999). Regardless of the marker’s shape, the GA phantom ensured visibility even when the tube current was reduced to the minimum value (10 mA). The low contrast resolution was gradually decreased at less than 50 mA, but the spatial resolution did not change. Although the auto‐registration function could not be used, manual‐registration could be achieved with an accuracy of within 1 mm, even when the imaging dose was reduced to 1/20 of the clinical imaging condition for the pelvis. When using the GA as the fiducial marker, the imaging dose could be reduced to 1/20 of that used clinically while maintaining the accuracy of manual‐registration using the kV‐FM for image‐guided radiotherapy of the pelvis.

## 1 INTRODUCTION

The high‐precision radiation therapy techniques, such as intensity‐modulated radiotherapy (IMRT), volumetric‐modulated arc therapy (VMAT), stereotactic body radiation therapy (SBRT), and image‐guided radiotherapy (IGRT) has been used commonly at many institutions. These techniques require high geometric accuracy and reproducibility of patient positioning. This requirement can increase the imaging frequency of megavoltage or kilovoltage planar imagings and cone‐beam CT (CBCT), among others. IGRT is problematic, however, because the normal tissue around the target is subjected to a high radiation dose due to the increased imaging dose as well as the treatment dose.^1^, ^2^ Furthermore, imaging fields are much larger than the radiotherapy field, so the imaging dose exposes tissue outside the radiotherapy field to unwelcome and unnecessary radiation. Epidemiological studies have shown increased skin cancer risk outside the radiotherapy field, even at relatively low skin doses.^3^ Therefore, it is necessary to find a way to reduce the imaging dose during IGRT as much as possible while ensuring the accuracy of image registration.^4^

The methods available for the reduction of the imaging dose include reducing the milliampere‐seconds (mAs) value, the projection number used for reconstruction, and the scan length, the use of a filter, and changing the imaging angle.^5^, ^6^, ^7^, ^8^, ^9^, ^10^, ^11^ Reducing the imaging dose, however, increases noise, which leads to deterioration in image quality.^12^ Gold fiducial markers can be used as a substitute to mark the position of the target tissue, which allows soft tissue matching.^13^ Planar kilovoltage orthogonal imaging with fiducial markers (kV‐FM) have been proved to afford better reproducibility of image registration and less intraobserver error than soft tissue matching with CBCT.^14^ In addition, the kV‐FM reduced the imaging dose and shortened the time required for image acquisition and position verification.^14^ Nevertheless, the visibility and registration accuracy of the kV‐FM images with reduced imaging doses has not been reported.

The purpose of this study was to evaluate the possibility of reducing the imaging dose used during IGRT by using the low tube current with kV‐FMs. The visibility of the fiducial marker, several image quality aspects, and the resultant registration accuracy were evaluated using variously shaped fiducial markers.

## 2 MATERIALS AND METHODS

### 2.A Gold fiducial markers

In this study, we used a Gold Anchor™ [(GA); Naslund Medical AB, Huddinge, Sweden] as the fiducial marker. The GA contains 0.5% iron, is associated with few artifacts, and is not only used for kV planar imaging, it provides excellent visibility with CBCT and magnetic resonance imaging for prostate and liver imaging clinically.^15^, ^16^ At least three fiducials are recommended to allow triangulation and measurement of the patient’s position in different planes.^13^ GAs come in various shapes, including a tadpole‐like shape, which provides image registration in three dimensions, thereby allowing the use of just one fiducial marker. In addition, the GA with 0.28 mm in diameter and 10 mm in length can be inserted with needles as thin as 25 gauge.^15^ Bleeding and pain are thus minimal, if not avoided, by using such a thin needle.

### 2.B Visibility evaluation of the GA

**Fig. 1:**
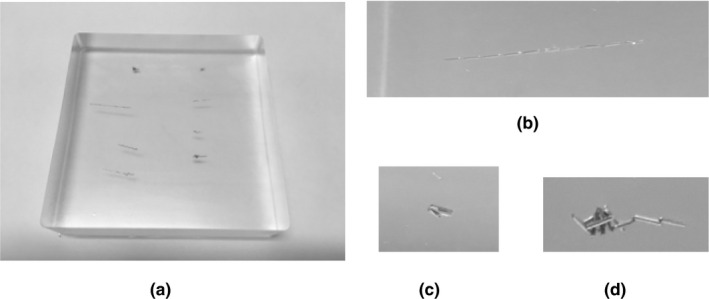
Shapes of a fiducial marker containing 0.5% iron [Gold Anchor™ (GA)]. (a) GA phantom. (b) GA in a linear shape. (c) GA in a folded shape. (d) GA in a tadpole‐like shape.

To evaluate the difference in visibility of the variously shaped GAs, a GA phantom was created by placing GAs of eight shapes (linear shape: 4‐, 8‐, 9‐, 13‐, and 18‐mm long; folded shape: 2‐ and 3‐mm long; tadpole‐like shape: 5‐mm long). Each was placed in the agar phantom (9.8 × 9.8 × 2.5 cm^3^) (Fig. 1).

**Fig. 2:**
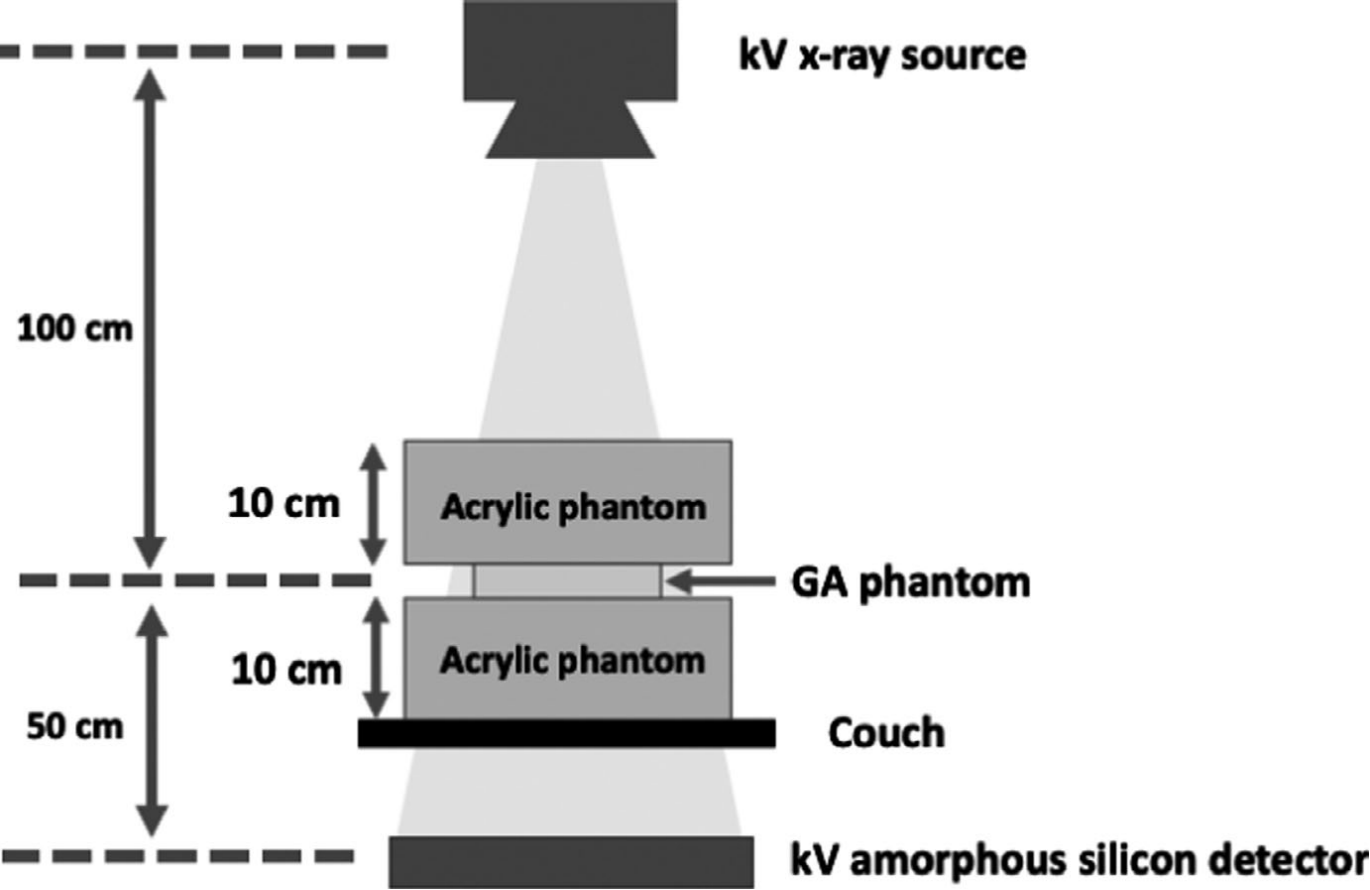
Measurement geometry for visibility evaluation of Gold Anchors^TM^.

Figure 2 shows the measurement geometry. The GA phantom was sandwiched between upper and lower acrylic phantoms with thicknesses of 10 cm. We tested the kilovoltage planar imaging (kV imaging) using the Clinac iX (Varian Medical Systems, Palo Alto, CA, USA) under clinical imaging conditions for the pelvis (75 kVp, 200 mA, 50 ms). We reduced the imaging dose by reducing the tube current (from 200 to 10 mA).

We also confirmed the visibility on digitally reconstructed radiographs (DRRs) of the planning CT (pCT), which was considered as the reference image for image registration. The pCT images of the GA phantom were obtained on a CT scanner (Alexion; Toshiba, Tokyo, Japan). The parameters included a X‐ray voltage of 120 kVp, X‐ray current of 200 mA, and helical pitch of 11.0. A DRR was obtained from the radiation treatment planning system (Eclipse version 11.0; Varian Medical Systems, Palo Alto, CA, USA) as the reference image. The slice thicknesses of the reconstructed pCT were 1, 2, and 3 mm, and the phantom was set at 0°, 45°, and 90° to exclude a partial volume effect.

### 2.C Measurement of imaging dose

The kV imaging doses were measured using a commercial semiconductor detector (NOMEX Multimeter, PTW, Freiburg, Germany) positioned at the front of acrylic phantoms under the same conditions as the visibility evaluation of the GA. The measurement uncertainty in the NOMEX is ±2.5% at the 95% confidence level. NOMEX is a non‐connected and compact multiparameter measuring device, which can be used simply for evaluating the effect of imaging dose reduction.^11^

### 2.D Evaluation of image quality

**Fig. 3:**
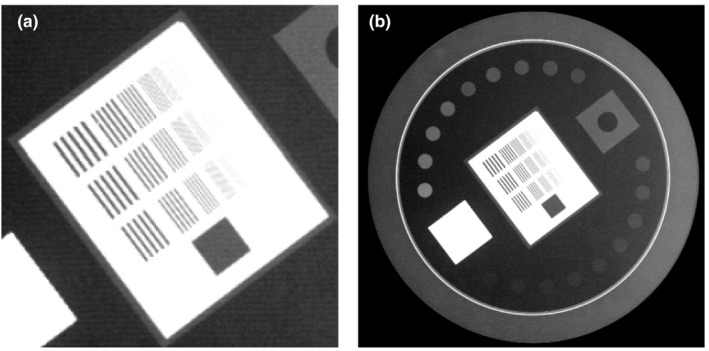
Sample images obtained with the TOR 18FG system for the image quality test. (a) Spatial resolution. (b) Low contrast resolution.

Image quality aspects of the kV images, including spatial resolution and low contrast resolution, were evaluated to determine the image quality with a TOR 18FG apparatus (Leeds Test Objects Ltd, North Yorkshire, UK) (Fig. 3). We placed the TOR 18FG on the cover of the kV amorphous silicon detector (kVD) with the kVD positioned at (−50, 0, 0). The blades were set at a 14 × 14 cm^2^ opening, and a 1‐mm copper plate was placed over the kV X‐ray source (kVS) to simulate a modestly thick patient.^17^ The evaluation methods for each aspect of image quality were as follows;^18^

- Twenty‐one bar patterns ranging between 0.50 and 5.00 lp/mm were used to evaluate the spatial resolution according to the smallest identifiable bar group visible in the image. The acceptable specification level of the On‐Board Imager (OBI) acceptance test was 1.25 lp/mm.^19^
- Eighteen disks of 8‐mm diameter, each with contrasts ranging between 16.7% and 0.9%, were used to evaluate visibility at low contrast resolutions according to the lowest contrast disc visible. The acceptable specification level of the OBI test was 2.33%.^19^

### 2.E Reproducibility of image registration

We confirmed the consistent shift results with that of the baseline protocol of image registration between the DRRs obtained via the pCT and the kV‐FM using various tube currents. The image registration was focused on each shape type of GA. Auto‐registration was performed using Offline Review image registration software (Varian Medical Systems, Palo Alto, CA, USA). The isocenter of the reference image was moved 5 mm along each of the two axes (superior/inferior and left/right) on the software, in order to eliminate the uncertain shift value by couch moving and evaluate only the effect of the low‐dose image on the image registration result. Radiation therapists who were well experienced in clinical site (more than 5 years) performed the manual‐registrations. Image registration was performed by looking at only the images with blinding as to amount of shift. Auto‐ and manual‐registrations were each performed three times for all exposure conditions. The registrations between the pelvic image using preset condition and the DRR were set as the reference value. The reproducibility of image registrations was evaluated according to differences in the translations along the two axes (superior/inferior and left/right) in comparison with the reference value.

## 3 RESULTS

### 3.A Visibility evaluation of the GA

**Fig. 4:**
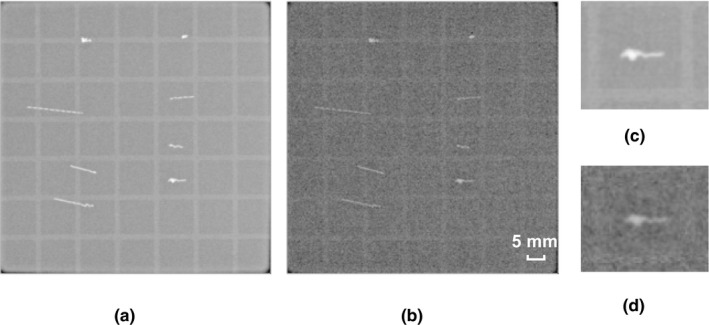
Visibility on kilovoltage (kV) images of the Gold Anchor™ (GA) phantom at (a) 200 mA and (b) 10 mA. Visibility of GA in a tadpole‐like shape at (c) 200 mA and (d) 10 mA.

**Fig. 5:**
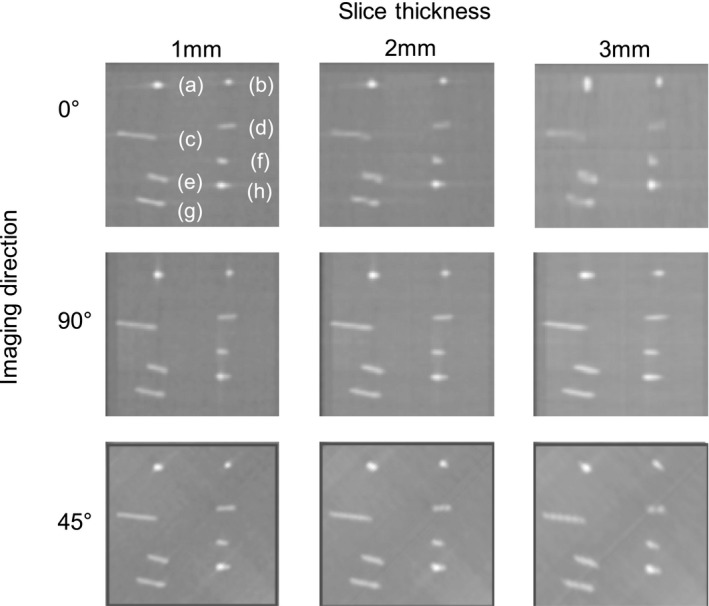
Visibility on digitally reconstructed radiographs of the Gold Anchor™ (GA) phantom with slice thicknesses of 1, 2, and 3 mm and the imaging directions rotated 45° and 90°. (a, b) GA in a folded shape. (c–g) GA in a linear shape. (h) GA in a tadpole‐like shape.

Figure 4 shows images of the GA phantom at 200 and 10 mA. Regardless of the shape of the marker, the GA phantom ensured visibility even when the milliamperage was reduced to the minimum value (10 mA). On DRRs, the tadpole‐like shaped GA was slightly recognized. In addition, when the slice thickness was ≥2 mm, the shape of the GA was extended in the slice thickness direction, making it difficult to recognize the shape (Fig. 5).

### 3.B Imaging dose

**Fig. 6:**
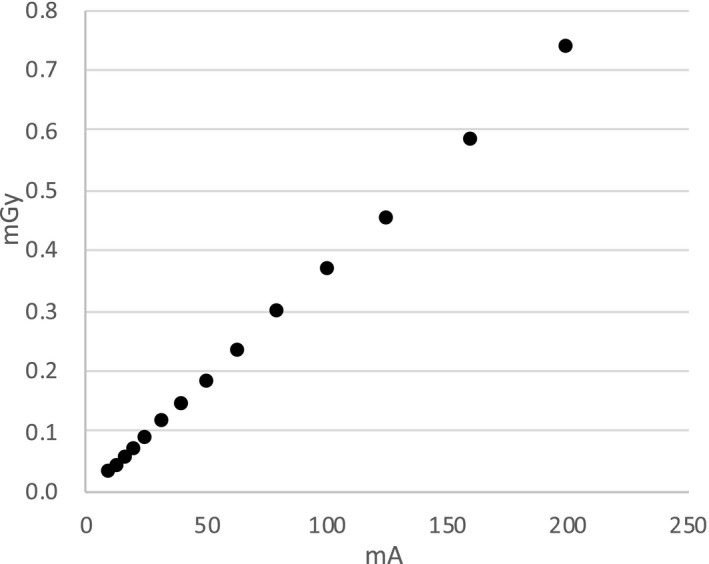
The absorbed doses (imaging doses) for kilovoltage planar imaging measured by NOMEX at the front of the acrylic phantom.

The imaging doses under each condition are shown in Fig. 6. The imaging doses for the preset condition and minimum dose condition (10mA) were 0.74 ± 0.00 mGy and 0.04 ± 0.00 mGy, respectively. A linear relationship between the mA values and imaging doses measured with the NOMEX was observed under each exposure condition (coefficient of determination: R^2^ = 0.999). The standard deviation (1SD) of the measurement by NOMEX was less than 1.0%.

### 3.C Image quality

**Fig. 7:**
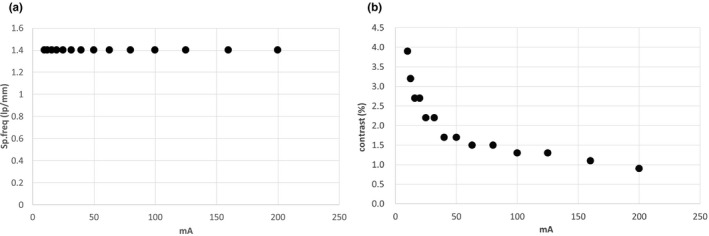
Results of (a) spatial resolution and (b) low contrast resolution.

The results of the image quality assessment are shown in Fig. 7. The spatial resolution did not change even when the milliamperage was reduced [Fig. 7(a)]. However, the low contrast resolution gradually deteriorated and degraded sharply at less than 50 mA [Fig. 7(b)]. Moreover, at ≤20 mA, it became worse than the OBI test’s acceptable specification level (<2.33%).

### 3.D Image registration

**Fig. 8:**
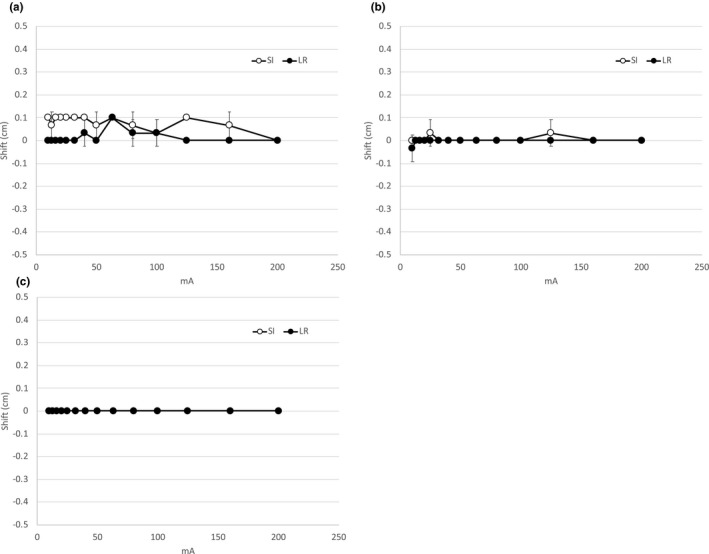
Results of manual image registration with Gold Anchors™ of various shapes. (a) Folded shape. (b) Linear shape. (c) Tadpole‐like shape. Error bars represent one standard deviation.

Although the auto‐registration function could not be performed, manual‐registration was possible even when the milliamperage was reduced to 10 mA. Image registration was possible within 1 mm regardless of the GA’s shape (Fig. 8). On DRRs with slice thicknesses of 2 and 3 mm, the visibility of the GA deteriorated, and image registration was impossible.

## 4 DISCUSSION

We evaluated the possibility of reducing the imaging dose during IGRT by using kV planar imaging with the GA as the fiducial marker. When we gradually decreased the contrast resolution, the spatial resolution did not change even when the milliamperage was reduced, thereby maintaining the visibility of the GA. We showed that image registration using GA could be achieved with an accuracy of within 1 mm using manual‐registration, even when the imaging dose was reduced to 1/20 that of the clinical imaging condition for the pelvis (Figs. 6 and 8). The implant fiducial markers using the optimization of the imaging dose during treatment without reduction of the amount of information in the image are beneficial for IMRT/VMAT and SBRT in patients with lung, liver, and prostate cancers.^16^, ^20^, ^21^ This is because the markers can be visualized on planar kV image clearly, even in soft tissue, as shown in Fig. 4. American Association of Physicists in Medicine Task Group 75 described the imaging dose can be concentrated at the skin and can be associated with deterministic skin injury, particularly in kV imaging.^4^ On the other hand, the association between risk (including cancer and genetic defects) and imaging dose is complex because concomitant doses are administered by both the therapeutic beam and imaging procedure. However, the International Commission on Radiological Protection 2007 Recommendation describes a simple proportionality between a few tens of mGy and cancer risk. It also mentions that the possibility of this relationship cannot be excluded even at doses below a few tens of mGy.^22^ Therefore, the cumulative imaging dose of planar kV images is not negligible, although results of this study show that the imaging dose per shot is approximately 1 mGy or less (Fig. 6). Attention should be paid to the risk/benefit balance because the visualization is highly individualized to the treatment site and protocol.^4^ This balance should be optimized carefully to minimize the risk as much as possible.

In this study, the reproducibility of image registration was evaluated using single planar image. In clinical image registration, three‐dimensional image registration is performed using orthogonal imaging. Since we performed image registration using various types of markers, it could be possible to evaluate the visibility of markers and the reproducibility of image registration using only single planar image. We tested various shapes of GA and found that shape itself did not affect their visibility despite the reduction in the imaging dose. With conventional spherical and rod‐like markers, three or more markers must be in place to perform three‐dimensional position matching.^13^ In contrast, the tadpole‐like GA could reduce the number of indwelling markers, as its shape provided multiple assessable points at once with a single marker. Reducing the number of indwelling markers also reduces the risk of complications while indwelling. The shape of the tadpole was visible even when the imaging dose was reduced to 1/20 of the clinical imaging condition (Fig. 4).

Although auto‐registration could not be performed because the GA was so small, the visibility could be ensured even at 10 mA (the lowest level), so manual‐registration by experienced radiation technicians was possible with a reproducibility within 1 mm, as shown in Fig. 8. This level of accuracy can be tolerated in consideration of the planning target volume margin. Low‐dose kV‐FM is an image registration method with a short image acquisition time, low imaging dose, and high registration accuracy.

This study has several limitations, including image quality on DRRs and use of the acrylic phantom, as patients are not rigid and vary in physique. Poor‐quality DRRs may not allow adequate verification of patient positioning because of the inability to visualize anatomical details. Geometrically inaccurate DRRs cause mistakes in the patient setup and treatment due to positioning errors.^23^ The visibility of the GA in DRRs used as a reference image for image registration is less clear than kV‐FM. When the slice thickness was ≥2 mm, the shape of the GA extended in the slice thickness direction, and the shape of the tadpole type could not be recognized (Fig. 5). It is necessary to insert multiple markers in order to perform three‐dimensional position matching with a slice thickness of 2 mm or more. If the priority is to reduce the number of inserted markers, the pCT slices thickness should be 1 mm. Furthermore, the contouring of the markers on each pCT slice during treatment planning enables clear identification of the markers on DRRs. It is generally understood that smaller slice thicknesses and spacing produces better spatial resolution of DRRs, but a slice thickness of 1 mm can only be justified for SBRT treatments, and it cannot be justified for many other sites. When using a slice thickness of 1 mm, such as in IMRT, several issues need to be addressed with the extra dose from higher resolution CT, longer scan times, the need for larger imaging storage solutions and marker distortion in DRR due to free‐breathing or average 4DCT. We tested various shapes of GA and found that the recognition of the GA did not depend on the shape and orientation which might be changed by marker migration, tumor change (shrinks or grows), surrounding tissue change, and patient rotation (yaw/pitch/roll) as a patient setup issue despite the reduction in the imaging dose. On the other hand, the physique of the patient can cause variations of image quality, which may make it impossible to perform accurate registration. It is also necessary to confirm the visibility of the GA when there is overlapping of organs with high CT values (e.g., bones). This study validated the usefulness of fiducial markers with lower imaging dose in radiotherapy by using the acrylic phantom as a first step and showed the possibility of specific dose reduction. For the next step, further study and careful verification in clinical situation needs to show this method is clinically useful.

## 5 CONCLUSION

When using the GA as a fiducial marker, the imaging dose could be reduced to 1/20 of the clinical imaging protocol for the pelvis while maintaining the accuracy of manual‐registration by kV‐FM, regardless of the shape of the GA.

## CONFLICT OF INTEREST

There is no conflict of interest.

## AUTHORS' CONTRIBUTIONS

Y Takei, H Monzen, and H Doi were involved in concept and design. Y Takei and M Tamura were involved in measurements and data analysis. Y Takei, H Monzen, M Tamura, H Doi, and Y Nishimura were involved in manuscript preparation. All authors read and approved the final manuscript.
